# Genotyping and In Silico Analysis of Delmarva (DMV/1639) Infectious Bronchitis Virus (IBV) Spike 1 (S1) Glycoprotein

**DOI:** 10.3390/genes13091617

**Published:** 2022-09-09

**Authors:** Ahmed Ali, Davor Ojkic, Esraa A. Elshafiee, Salama Shany, Mounir Mohamed EL-Safty, Adel A. Shalaby, Mohamed Faizal Abdul-Careem

**Affiliations:** 1 Faculty of Veterinary Medicine, University of Calgary, 3330 Hospital Drive NW, Calgary, AB T2N 4N1, Canada; 2 Department of Pathology, Faculty of Veterinary Medicine, Beni-Suef University, Beni Suef 62511, Egypt; 3 Animal Health Laboratory (AHL), University of Guelph, Guelph, ON N1G 2W1, Canada; 4 Department of Zoonoses, Faculty of Veterinary Medicine, Cairo University, Giza 12211, Egypt; 5 Department of Poultry Diseases, Faculty of Veterinary Medicine, Beni-Suef University, Beni Suef 62511, Egypt; 6 Central Laboratory for Evaluation of Veterinary Biologics (CLEVB), Abassia, Cairo 11517, Egypt

**Keywords:** infectious bronchitis virus (IBV), spike 1 (S1) gene, phylogeny, Delmarva (DMV/1639), bioinformatic analysis

## Abstract

Genetic diversity and evolution of infectious bronchitis virus (IBV) are mainly impacted by mutations in the spike 1 (S1) gene. This study focused on whole genome sequencing of an IBV isolate (IBV/Ck/Can/2558004), which represents strains highly prevalent in Canadian commercial poultry, especially concerning features related to its S1 gene and protein sequences. Based on the phylogeny of the S1 gene, IBV/Ck/Can/2558004 belongs to the GI-17 lineage. According to S1 gene and protein pairwise alignment, IBV/Ck/Can/2558004 had 99.44–99.63% and 98.88–99.25% nucleotide (nt) and deduced amino acid (aa) identities, respectively, with five Canadian Delmarva (DMV/1639) IBVs isolated in 2019, and it also shared 96.63–97.69% and 94.78–97.20% nt and aa similarities with US DMV/1639 IBVs isolated in 2011 and 2019, respectively. Further homology analysis of aa sequences showed the existence of some aa substitutions in the hypervariable regions (HVRs) of the S1 protein of IBV/Ck/Can/2558004 compared to US DMV/1639 isolates; most of these variant aa residues have been subjected to positive selection pressure. Predictive analysis of potential N-glycosylation and phosphorylation motifs showed either loss or acquisition in the S1 glycoprotein of IBV/Ck/Can/2558004 compared to S1 of US DMV/1639 IBV. Furthermore, bioinformatic analysis showed some of the aa changes within the S1 protein of IBV/Ck/Can/2558004 have been predicted to impact the function and structure of the S1 protein, potentially leading to a lower binding affinity of the S1 protein to its relevant ligand (sialic acid). In conclusion, these findings revealed that the DMV/1639 IBV isolates are under continuous evolution among Canadian poultry.

## 1. Introduction

Avian infectious bronchitis (IB) represents one of the globally ubiquitous diseases of commercial poultry, characterized by high morbidity and variable mortality, and with subsequent economic losses [1]. IB is induced by an infectious bronchitis virus (IBV) that is a part of the genus *Gammacoronavirus*, the family *Coronaviridae*, and the order *Nidovirales* [2]. IBV possesses a single-stranded positive-sense ribonucleic acid (RNA) genome with a length of around 27.6 kb, and the two untranslated regions (UTRs) are located at its 5′ and 3′ ends [3,4]. The genome has a minimum of 10 open reading frames (ORFs) arranged as 5′-1a-1b-spike (S)-3a-3b-envelope (E)-membrane (M)-5a-5b-nucleocapsid (N)-3′ [5]. A large polyprotein 1ab, responsible for RNA replication and transcription, is translated from two overlapping ORFs (1a, 1b) that constitute two-thirds of the genome. Four major structural proteins (S, E, M, and N) are encoded by the rest of the genome, and there are two accessory genes (3 and 5) translated into four accessory proteins (3a and 3b, 5a and 5b, respectively) [6].

The S glycoprotein is one of the main structural proteins, and it is post-translationally cleaved by proteolysis into S1 and S2 subunits at the furin consensus specific site (RRFRR/HRRR) to enhance virus binding to receptor and entry into the host cell [7]. There are three hypervariable regions (HVRs): HVR 1 (amino acid (aa) position 38–67), HVR 2 (aa 91–141), and HVR 3 (aa 274–387), located in the S1 subunit [8,9]. The HVRs play important roles in viral infectivity by possessing a minimum of five neutralization sites [10] and serotype-specific epitopes [11]. HVRs vary widely, resulting in an array of antigenic variants around the world [12]. The S1 subunit is most likely associated with high mutation rates and recombination events [13,14,15,16]. The S2 glycoprotein includes an ectodomain, a transmembrane part, and a C-terminal domain, and its function is to facilitate the virus fusion to the host cell membrane [7]. Several studies have revealed that the S1 subunit has a receptor-binding domain (RBD) that includes many aa residues that can fold in an independent manner, and they are crucial for virus attachment to host cell receptors such as sialic acid [17,18,19].

Genetic categorization of IBV variants is commonly performed by phylogenetic analysis of a complete sequence of the S1 gene, encoding the relevant protein. There are seven genotypes (GI-GVII), including 35 genetic lineages according to phylogenetic analysis of the IBV S1 gene [20]. Most of the genetic lineages (29 lineages) are related to Genotype GI, and each of the remaining genotypes contains only one lineage. A majority of IB endemic countries have IBV from the Massachusetts (Mass) lineage (GI) and the 793B lineage (GI-12), whereas the other lineages spread only in certain regions throughout the world [21,22]. The lineage GI-17 involves IBV isolates in the USA, which were linked to respiratory, renal, and reproductive diseases [22]. In 2011, multiple nephropathogenic IB outbreaks in the Delmarva peninsula, USA, have been associated with a new variant of IBV known as Delmarva (DMV)/1639, which belongs to GI-17 [23]. Since 2015, there has been an increasing prevalence of IBV strains genetically identical to the DMV/1639 variant in poultry operations in eastern Canada [24]. It has been shown that the Canadian DMV/1639 strain induces marked pathological lesions in different body systems, including respiratory, renal, and reproductive systems [25].

The N-glycosylation of viral protein plays a key role in virus virulence and tissue tropism [26]. The glycosylation sites in the M and S proteins of coronaviruses could participate in viral fusion, receptor binding, and antigenic properties [27,28,29,30]. Changes in the N-glycosylation motifs of viral proteins could impact receptor binding, thus leading to lower recognition efficiency by host innate immune responses and antibodies, eventually affecting viral infectivity and replication [31,32,33]. Phosphorylation of proteins has essential advantages for viral assembly and replication as it helps in the modulation and regulation of physiological functions of virus proteins [34,35]. Palmitoylation shares numerous functions with viral proteins such as sub-cellular localization and transport and protein–protein interactions, in addition to different physiological features [36,37]. In the case of coronaviruses, palmitoylation has an impact on viral glycoproteins relevant to fusion to cellular membranes, viral assembly, and infectivity [38,39].

The majority of genomic variance can be attributed to single nucleotide polymorphisms (SNPs) [40], which are greatly associated with phenotypic changes and multiple diseases. Missense SNPs are varieties of non-synonymous SNPs (nsSNPs); they cause aa substitutions and lead to damaging or neutralizing effects. Various types of computational tools have been recognized to detect the potential SNPs and predict their mutational impacts on the protein function and structure [41,42,43,44,45,46]. These tools are reliable, easy to use, fast, and of low cost [47]; therefore, they could be applied as preliminary steps to filter the potential deleterious nsSNPs before performing the experimental screening to detect the mutations. The present study aimed to analyze the whole genome sequence of IBV/Ck/Can/2558004 isolate with special emphasis on the prospective effects of aa changes on the function and structure of the S1 glycoprotein by predictive bioinformatic tools.

## 2. Materials and Methods

### 2.1. Virus Isolation and Propagation

The IBV isolate, IBV/Ck/Can/2558004, was isolated from one clinical sample obtained from the Animal Health Laboratory (AHL), at the University of Guelph, Canada. The examined sample was collected from two-week-old broiler chickens raised in a commercial farm with a capacity of 46,000, situated in Ontario, Canada. For virus isolation and propagation, embryonated chicken-specific pathogen-free (SPF) eggs were purchased from the Canadian Food Inspection Agency (CFIA), Ottawa, Ontario, Canada. The embryonated chicken eggs were kept in the incubator at 37 °C until the embryos were 9 to 11 days old; afterwards, they were inoculated with 200 µL of sample supernatant into the allantoic cavity. Eggs that had embryo deaths after 24 h of incubation were eliminated from further analysis. The allantoic fluid was harvested 2 days following inoculation and then aliquoted and stored at −80 °C. Serial passage of the obtained allantoic fluid was performed in the embryonated eggs two times to increase the virus titer.

### 2.2. RNA Extraction and Whole Genome Sequencing

The viral RNA was extracted from 200 µL of allantoic fluid using a Quick-RNA Viral Kit (ZymoResearch, Irvine, CA, USA, Catalog # R1034), according to the manufacturer’s instructions. The concentration of resulted RNA was measured by a Nanodrop1000 spectrophotometer (ThermoScientific, Wilmington, DE, USA), based on absorbance at a 260/280 nm wavelength. The complementary DNA (cDNA) was converted from 150 ng of the obtained RNA using random primers (High-Capacity Reverse Transcription Kit™, Applied Biosystems, Invitrogen Canada Inc., Burlington, ON, Canada), as indicated by the manufacturer. To detect and confirm IBV propagation on embryonated eggs, an already established real-time PCR was performed, targeting the IBV-N gene [48]. The RT-qPCR positive samples with Cq lower than 20 was submitted to the whole genome sequencing using a Miseq platform (Illumina corp, San Diego, CA, USA) at the Faculty of Veterinary Medicine, University of Montreal, Montreal, Quebec, Canada.

### 2.3. Phylogenetic Analysis and Pairwise Alignment of S1 Gene and Protein

The phylogenetic analysis and genetic relatedness were based on the complete nucleotide (nt) sequence of the S1 gene of IBV/Ck/Can/2558004 and 85 reference IBV strains (Table S1) retrieved from GenBank (https://www.ncbi.nlm.nih.gov/genbank/) (accessed on 1 February 2022). The retrieved reference sequences were considered to be representative for each genotype and lineage as previously described [22]. We performed the pairwise alignment of S1 nt sequences using fast Fourier transformation (MAFFT) [49] as a tool built into the Geneious^®^ software v10.2.6 (https://www.geneious.com/) (accessed on 5 February 2022). The phylogenetic analysis was constructed by maximum-likelihood method using 1000 bootstrap replicates in MEGA X software [50]. Then, the resulting tree was edited and visualized using the iTOL v4 program [51]. MAFFT was also employed to align the aa sequences of the S1 protein of IBV/Ck/Can/2558004 and other Canadian and US DMV/1639 IBVs (Table S2).

### 2.4. Prediction of Post-Translational Modifications Based on S1 Glycoprotein

S1 glycoprotein of IBV/Ck/Can/2558004 and MDL_DMV1639_15-5582 (KX529725) isolates were subjected to post-translational modifications analysis. Firstly, the N-glycosylation sites were predicted with the aid of a web server called NetNGlyc-1.0 [52] (https://services.healthtech.dtu.dk/service.php?NetNGlyc-1.0) (accessed on 14 February 2022). For recognition of potential phosphorylation sites, the NetPhos-3.1 [53] (https://services.healthtech.dtu.dk/service.php?NetPhos-3.1) (accessed on 20 February 2022) was used. The palmitoylation sites were determined by CSS-Palm 4.0 software (http://csspalm.biocuckoo.org/) (accessed on 20 February 2022), which depends on the fourth generation of the Group-based Prediction System (GPS) algorithm [54].

### 2.5. Identification of Codon Sites under Selection Pressure in S1 Glycoprotein

To find the aa residues under selection pressure, the aa sequence alignment of S1 glycoproteins of eight DMV/1639 IBVs (Table S2) and IBV/Ck/Can/2558004 were examined in the SELECTION server (https://selecton.tau.ac.il/) (accessed on 10 February 2022). For every single site within the protein sequence, the SELECTION server could count the ratio between synonymous (dS) and non-synonymous (dN) substitutions [55]. Additionally, the obtained ratio is shown on a colored scale plot sorting the selection into positive, neutral, or purifying.

### 2.6. In Silico Prediction Analysis on Functional, Structural Impacts, and Interatomic Interactions

Initially, we employed aa sequence alignment for both S1 glycoproteins of IBV/Ck/Can/2558004 and MDL_DMV1639 _15-5582 (KX529725) to list the aa substitutions using MAFFT. To predict the functional outcomes of aa substitutions, PROVEAN, SIFT, Poly-Phen-2, PhD-SNP, and SNPs&GO were performed.

PROVEAN (http://provean.jcvi.org/index.php) (accessed on 20 February 2022), an online database, expects the deleterious effects of single or numerous aa changes on protein function [56]. The variant codon can be deleterious when its PROVEAN score is ≤−2.5. The SIFT tool (https://sift.bii.a-star.edu.sg/) (accessed on 20 February 2022) anticipates the capability of the mutant codons within the protein to affect the function through sequence homology and physical properties of protein residues [57]. The prospective functional effects of aa changes within the protein were evaluated using Poly-Phen-2 (http://genetics.bwh.harvard.edu/pph2/) (accessed on 22 February 2022) based on reasonable physical and comparative foresights [58]. The obtained data by Poly-Phen-2 were categorized into possibly damaging (probabilistic score >0.15), probably damaging (probabilistic score >0.85), and benign (remaining). PhD-SNP (https://snps.biofold.org/phd-snp/phd-snp.html) (accessed on 22 February 2022) is a web server that depends on the support vector machines to predict the disease-related effects of aa variations within the protein [59]. Based on the function of protein annotation, SNPs&GO (https://snps.biofold.org/snps-and-go/snps-and-go.html) (accessed on 22 February 2022) is employed to ratify whether the variant codons have disease effects or not within the tested protein [60].

To predict the structural effects of aa changes, seven computational tools (MutPred2, MUpro, NetSurfP-2.0, DUET, mCSM, SDM, HOPE) were used. MutPred2 (http://mutpred.mutdb.org/) (accessed on 25 February 2022), an online application, depends on a probabilistic paradigm to discover the functional and structural effects of variants within the protein, and thus could be beneficial for the mutants associated with phenotype alteration in the experimental analyses [61]. MUpro (https://www.ics.uci.edu/~baldig/mutation.html) (accessed on 25 February 2022) relies on support vector machines to expect the effect of each point mutation on the protein stability with 84% accuracy [62]. NetSurfP-2.0 (https://services.healthtech.dtu.dk/service.php?NetSurfP-2.0) (accessed on 25 February 2022) was employed to anticipate the secondary structure, solvent accessibility, and structural changes among each codon site within the examined protein [63]. DUET, mCSM, and SDM (http://biosig.unimelb.edu.au/duet/) (accessed on 25 February 2022) are collectively implemented in the same web server and they depend on the support vector Machines [64]. Using the 3D structure of the protein as an input, the HOPE server (http://www.cmbi.ru.nl/hope/input/) (accessed on 27 February 2022) was performed to analyze the impacts of variable aa residues on protein function and structure [65].

For the aim of interatomic interactions analysis, the DynaMut server (http://biosig.unimelb.edu.au/dynamut/) (accessed on 1 March 2022) was employed to accomplish the consequences of mutant aa residues on the protein flexibility and stability [66].

### 2.7. Homology Modelling and Structure Verification of S1 Glycoprotein

Based on aa sequences of S1 glycoprotein of IBV/Ck/Can/2558004 and MDL_DMV1639 _15-5582 (KX529725) isolate, the I-TASSER server (http://zhanglab.ccmb.med.umich.edu/I-tasser.) (Accessed on 1 January 2022) was performed to generate their 3D structures. The I-TASSER depends on aa sequence, and the output includes secondary, tertiary, and 3D structures predictions, as well as some protein function-related purposes [67]. After homology modeling, a web server called COACH (http://zhanglab.ccmb.med.umich.edu/COACH/) (accessed on 1 January 2022) was used to recognize the active binding sites against specific ligands. The COACH server acts by two methods named TM-SITE and S-SITE [68]. The comparison and identity between the two 3D modeled structures were achieved using UCSF Chimera software (http://www.cgl.ucsf.edu/chimera/) (accessed on 4 January 2022). The quality of the generated structures was verified using the Ramachandran Plot Server (https://zlab.umassmed.edu/bu/rama/index.pl) (accessed on 4 January 2022), which shows a colored scale graph with three categories (highly preferred conformations, preferred conformations, questionable conformations).

### 2.8. Analysis of Protein–Ligand Interactions by Molecular Docking

Initially, the sialic acid 3D structure (PubChem CID 444885) was retrieved from the PubChem database (https://pubchem.ncbi.nlm.nih.gov/) (accessed on 11 January 2022) and was utilized as a ligand. Then, the ligand was prepared through minimizing the energy by Avogadro software [69]. Similarly, S1 proteins in PDB format were viewed by a Swiss PDB viewer for energy minimization [70]. Following protein and ligand preparations, the molecular docking was blindly employed using AutoDock Vina integrated within the CB-Dock server [71]. For visualization of the docked outputs, Discovery Studio was used to clarify the protein–ligand interactions [72].

## 3. Results

### 3.1. Characteristics of Whole Genome Sequence, Phylogenetic Analysis, and Comparative Sequence Alignment

The full genomic length of IBV/Ck/Can/2558004 was submitted to GenBank under accession number ON950740. The complete genome length was 27,710 nt, and it included 13 ORFs (5′UTR-1a-1b-S-3a-3b-3c(E)-M-4b-4c-5a-5b-N-6b-3′UTR). ORF1a and 1b consisted of 11,889 nt and 8034 nt, respectively, and they formed the gene 1. The S glycoprotein was encoded by gene 2, and it had 3501 nt (encoding 1166 aa). Furthermore, the S protein was cleaved post-translationally into the S1 and S2 subunits by cleavage site RRSRR into 536 aa and 625 aa residues, respectively. Gene 3 contained 3 ORFs (3a, 3b, 3c) that encoded three different-sized proteins (57 aa, 64 aa, 109 aa, respectively). The M protein was encoded by a single ORF consisting of 678 nt (225 aa) and was located in gene 4. while gene 5 included 4b and 4c ORFs that had 94 and 56 aa, respectively. Two ORFs (5a, 5b) were observed at gene 6, and they were composed of 198 nt and 249 nt with 65 aa and 82 aa, respectively. A single ORF (1230 nt) was located at gene 7 encoding the N protein with 409 aa. A non-coding region (8 nt) between gene N and 6b has been detected. The length of UTRs at both genomic ends, 5′ and 3′, were 530 nt and 274 nt, respectively.

**Figure 1:**
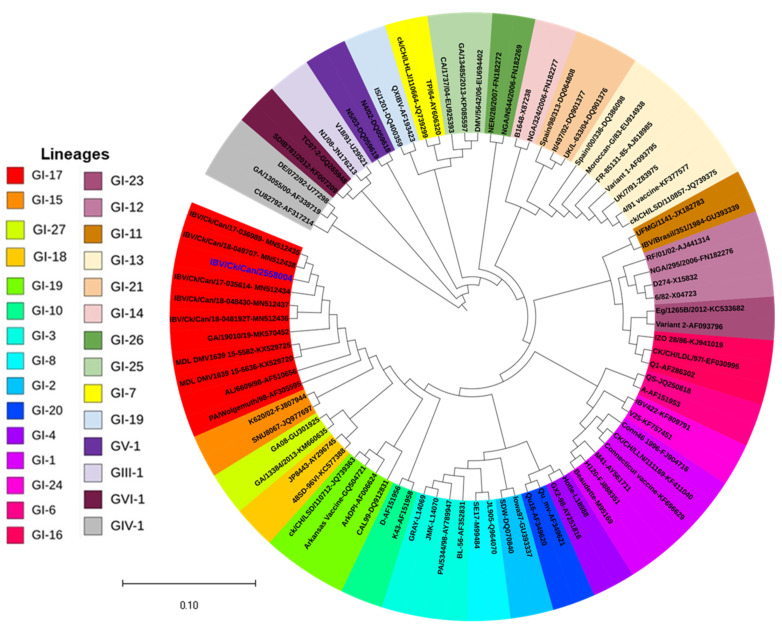
Phylogenetic relationships between IBV/Ck/Can/2558004 (denoted with blue color) and 85 reference strains based on the nt sequences of the S1 gene. The tree was constructed using the maximum-likelihood method and the Tamura–Nei model with 1000 bootstrap replicates in MEGA X software. Strains located within the same lineage are indicated in the same color. The constructed tree was viewed by iTOL software.

Based on S1 gene homology analysis, IBV/Ck/Can/2558004 had 99.63%, 99.50%, 99.44%, 99.50%, and 99.57% identities when compared to five Canadian DMV/1639 IBVs: IBV/Ck/Can/17-035614, IBV/Ck/Can/18-048430, IBV/Ck/Can/18-048192T, IBV/Ck/Can/17-036989, and IBV/Ck/Can/18-049707 [24], respectively. Moreover, IBV/Ck/Can/2558004 was related to the lineage GI-17 (Figure 1). Meanwhile, IBV/Ck/Can/2558004 shared 96.63% and 97.69% identities with MDL_DMV1639_15-5582 (KX529725) and GA9977/2019 (MK878536), respectively. The later IBV strains were related to DMV/1639, IBVs that were isolated in 2011 and 2019, respectively in the USA [23,73].

### 3.2. Pairwise Comparison Based on the aa Sequences of S1 Glycoprotein

The similarity of the deduced aa sequences of the S1 protein of IBV/Ck/Can/2558004, other Canadian DMV/1639, and USA DMV/1639 IBVs isolated between 2011 and 2019 are presented in Table S3. The S1 protein of IBV/Ck/Can/2558004 had 94.78% aa identity when compared to that of MDL_DMV1639 _15-5582 (KX529725), and there were 29 aa substitutions among IBV/Ck/Can/2558004, indicating that the nt changes were mostly of non-synonymous type. Two out of twenty-nine aa substitutions were located in HVR 1 at positions ^54^A and ^60^S, while six variants were observed in the HVR 2 at positions ^104^V, ^113^R, ^116^H, ^117^F, ^123^F, and ^136^I. HVR 3 included four aa changes at positions ^278^F, ^315^S, ^339^T, and ^369^N. Alternatively, the IBV/Ck/Can/2558004 S1 protein shared the highest aa similarity (98.88% to 99.25%) in comparison with that of the Canadian DMV/1639 isolates, and it had three aa substitutions. Two of the three aa variants were nearly situated in HVR 1 and HVR 2 at positions ^31^R and ^269^I, respectively, while the S1 protein of GA9977/2019 (MK878536) exhibited 97.20% aa identity in comparison with that of IBV/Ck/Can/2558004, where four aa changes were found in and/or near HVR 1, HVR 2, and HVR 3 at positions (^31^R, ^54^A,), (^91^S, ^95^T, ^116^H,), and (^269^I, ^293^T, and ^295^Q), respectively.

### 3.3. Prediction of Potential N-Glycosylation, Phosphorylation, and Palmitoylation Sites

A predictive computational analysis was performed to identify and compare post-translational modifications such as N-glycosylation, phosphorylation, and palmitoylation motifs in the S1 glycoprotein of IBV/Ck/Can/2558004 and that of MDL_DMV1639 _15-5582 (KX529725).

**Figure 2:**
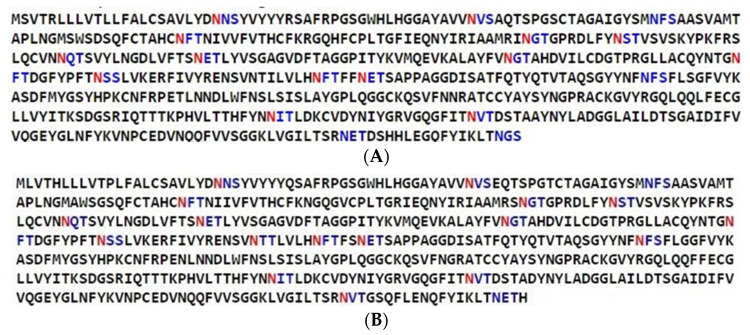
The sequons Asn-X-Ser/Thr in the S1 glycoprotein of IBV/Ck/Can/2558004 (**A**) and MDL_DMV1639 _15-5582 (KX529725) (**B**). Asn-Xaa-Ser/Thr sequons in the sequences are highlighted in blue. Sequons predicted to be N-glycosylated by NetNglyc server are shown in red.

N-glycosylation motifs were predicted based on a consensus sequence (sequon) Asn-X-Ser/Thr, where Asn refers to Asparagine, X refers to any aa except Proline (Pro), Ser is Serine, and Thr refers to Threonine. Among the S1 glycoprotein of IBV/Ck/Can/2558004, there were 41 sequons (Asn-X-Ser/Thr) where 14 sites were predicted to be N-glycosylated (Figure 2A), whereas the S1 glycoprotein of MDL_DMV1639 _15-5582 (KX529725) displayed 43 sequons, in which 17 were expected to have N-glycosylation (Figure 2B). Therefore, the IBV/Ck/Can/2558004 S1 protein lacks three N-glycosylation sites compared to that of MDL_DMV1639 _15-5582 (KX529725); one of the three sites was lost because the aa isoleucine (I) substituted threonine (T) at position 269.

The prospective phosphorylation sites were identified by the NetPhos 3.1 server, which predicts Ser, Thr, or tyrosine (Tyr) phosphorylation sites in the S1 glycoprotein. The conserved potential phosphorylation sites and their positions in the S1 glycoprotein of IBV/Ck/Can/2558004 and MDL_DMV1639 _15-5582 (KX529725) are listed in Table S4. S1 glycoprotein of IBV/Ck/Can/2558004 showed lack of Thr and Tyr phosphorylation sites at positions 62 T, 121 T, 372 T, 519 T, and 459 Y, respectively, in comparison with that of MDL_DMV1639 _15-5582 (KX529725). On the other hand, the IBV/Ck/Can/2558004 S1 protein exhibited acquisition of Thr and Ser phosphorylation sites at positions 101 T, 533 T, and 25 S, respectively, when compared to that of MDL_DMV1639 _15-5582 (KX529725). Ser substitutions at positions 2 S and 87 S added unique Ser phosphorylation sites to the conserved sites in IBV/Ck/Can/2558004.

To predict the palmitoylation sites, CSS-Palm 4.0 software was employed. The prospective palmitoylation sites were conserved and showed the same pattern in the S1 glycoprotein of IBV/Ck/Can/2558004 and that of MDL_DMV1639 _15-5582 (KX529725) (Table S5).

### 3.4. Selection Pressure Analysis of S1 Glycoprotein

**Figure 3:**
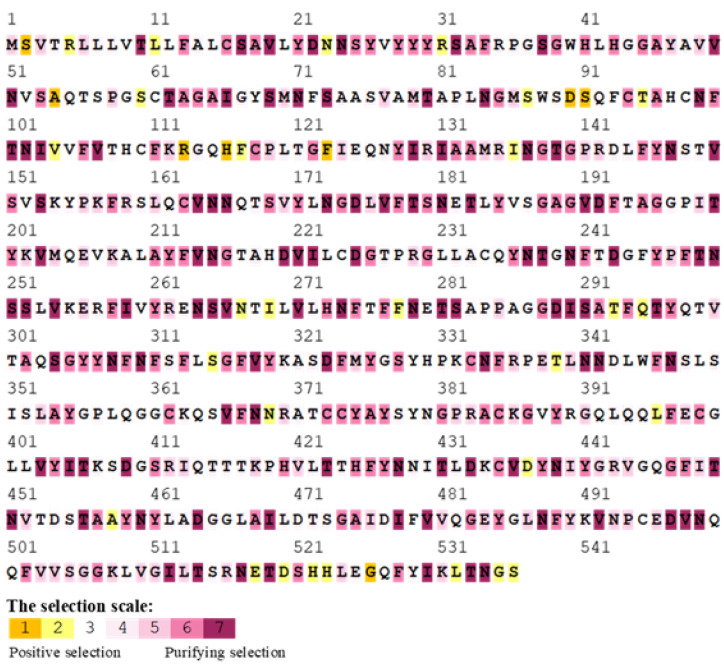
The MEC model in the SELECTION server was used to detect the selective pressure for the aa sequences alignment of S1 glycoprotein for 9 DMV/1639 IBVs, including IBV/Ck/Can/2558004. The aa residues colored with orange to yellow refer to a positive selection, while the residues highlighted with white and gray are indicative of a neutral selection. The purifying selection is represented by a purplish color.

The selection pressure examination was conducted by a web server called SELECTION. It estimates the selection pressure by using a Mechanistic Empirical Combination (MEC) model at certain codons. The MEC model acts by consideration of the different rates of aa changes. The positive selection was observed at different aa residues all over the S1 glycoprotein (Figure 3). The positive selection was detected in a total of 35 aa residues (6.5%), while the rest were subjected to purifying selection. Furthermore, it was shown that most aa residues subjected to positive selection were in and/or near the residues forming the three HVRs of S1 glycoprotein.

### 3.5. Impact of aa Substitutions on Function and Structure of S1 Glycoprotein

Because the highest aa changes in the S1 glycoprotein were detected when comparing IBV/Ck/Can/2558004 to MDL_DMV1639 _15-5582 (KX529725), we therefore considered the later as a wildtype (non-mutant). Further computational analysis was conducted on the wildtype S1 glycoprotein versus (vs.) IBV/Ck/Can/2558004 (mutant one) to study the prospective effects of aa substitutions on protein function, structure, and physico-chemical properties as well as to analyze interatomic interactions and protein–ligand interaction.

#### 3.5.1. Prediction of Functional Effect of aa Substitutions on S1 Glycoprotein

To detect the functional effect of aa substitutions on the S1 protein of IBV/Ck/Can/2558004, a total of five tools were used (PROVEAN, SIFT, Poly-Phen-2, PhD-SNP, SNPs&GO). Twelve out of twenty-nine aa substitutions were predicted by the SIFT server to have a damaging effect on the S1 protein, and their tolerance index was 0. The PROVEAN server showed four aa residues had a deleterious effect (PROVEAN score > −2.5). The number of affecting ones expected with the aid of PolyPhen-2 was five. The damaging variants identified by poly-Phen-2 were five; four of them were “probably damaging” (score 0.980–1.00) and only one was “possibly damaging” (score 0.919). The disease -associated variant aa residues predicted by PhD-SNP, SNAP-2, and SNPs&GO were nine, five, and one in number, respectively.

A total of eight variant aa residues were collectively anticipated by three or more tools to have damaging effects and were therefore functionally significant. These variants were taken into account in the following analyses.

#### 3.5.2. Prediction of Structural Impact of aa Substitutions on S1 Glycoprotein

Eight variants (L2S, Q31R, G116H, T269I, G520D, Q522H, F523H, N526G) that had been expected to cause a negative effect on protein function were then subjected to structural impact analysis. For this, seven tools (MutPred2, MUpro, NetSurfP-2.0, DUET, mCSM, SDM, HOPE) were used.

MutPred2 exhibited four mutants to be pathogenic, while Mupro predicted three variants to increase the stability of the protein structure, and the rest (L2S, G116H, T269I, F523H, N5226G) had a decreasing effect. NetSurfP-2.0 showed that the residue is either buried or exposed among the protein structure. It was shown that one mutant (T269I) shifted from exposed to buried in comparison with the wildtype S1 structure. Additionally, DUET predicted five mutants with destabilizing effects, and the others had stabilizing effects. In terms of SDM, four showed stabilizing effects, whereas mCSM revealed seven aa residues to be destabilizing. The HOPE server was used to determine the impact of physico-chemical characters, solvent accessibility, the interaction between the molecules, and effects on function and structure. Seven mutants were anticipated to induce a change in protein size, and three mutations revealed a modification in the charge. In the case of solvent accessibility, four mutations induced a variation in the hydrophobicity while two lost their hydrophobicity. Glycine residues at positions 116 and 520 of the wildtype structure were flexible enough to make torsion angles but replacing glycine with other aa residues caused the local backbone to be in an incorrect conformation, thus disrupting the local structure in the mutant S1 protein.

#### 3.5.3. Prediction of Inter-Atomic Interactions

**Figure 4:**
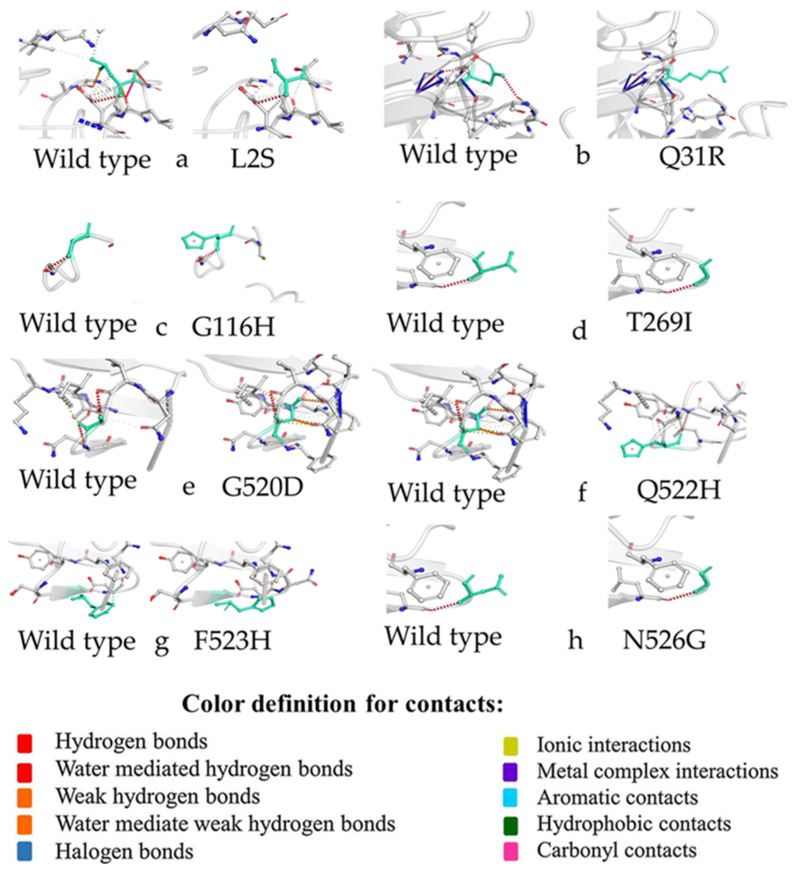
Prediction of interatomic interactions of the wildtype S1 protein vs. the mutant one using DynaMut. Non-mutant and variant aa residues (from **a**–**h**) are shown in light green and appear as sticks associated with the remaining residues forming the interaction. Dot points of several colors refer to the types of interactions.

The atomic interactions of the native and variant aa residues of the S1 protein were analyzed and illustrated by the DynaMut server (Figure 4). It has been observed that the ΔΔG EnCoM revealed all mutants to have a destabilizing effect. Three mutants (L2S, G116H, T269I) had an increased effect on molecule flexibility predicted by ΔΔSVib ENCoM. Meanwhile, the DynaMut ΔΔG predicted four mutants with stabilizing effects (Q31R, T269I, G520D, N526G).

#### 3.5.4. Homology Modelling and Quality Validation of the 3D Structures

**Figure 5:**
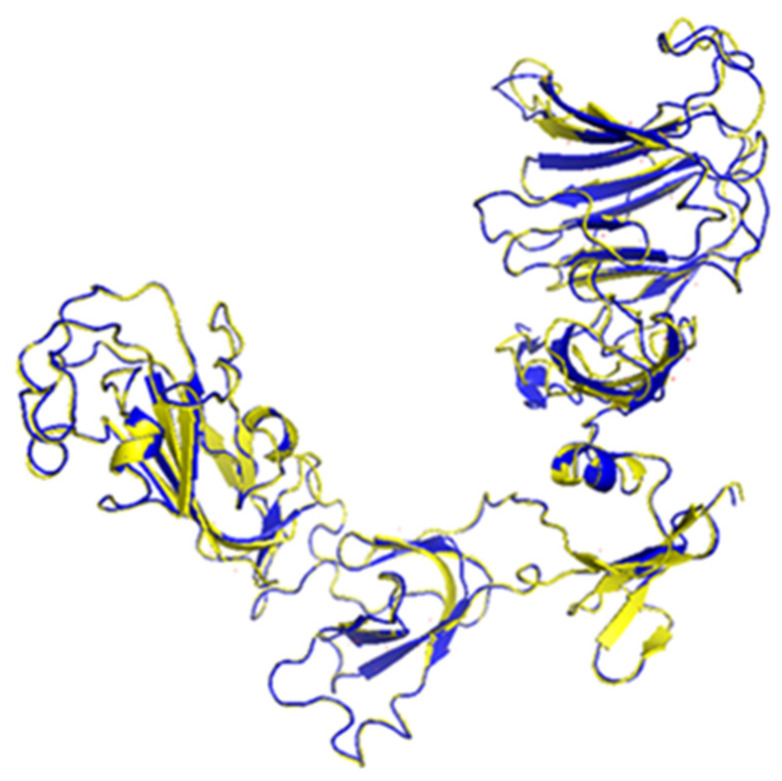
Homology modeling predicted by I-TASSER. 3D structures of S1 protein of wildtype (blue) and mutant (yellow) are shown.

To generate the 3D structures of the wildtype and mutant S1 protein of DMV/1639 IBVs, the I-TASSER server was employed. The generated models were topped in a certain arrangement and classified by the TM-score, which degrees the distance of deviation (in Angstrom) between the residual position of the model and wildtype structure. For both of the modeled sequences utilized in this study, the TM-score output exceeded 0.5, thus indicating that both 3D structure models were biologically significant and had a convenient structural topology (Figure 5; Table 1). The similarity between both 3D structures (wildtype and mutant S1 protein of DMV/1639 IBVs) was 88.43%. Apart from structural similarity, both 3D models showed common active binding sites to some exogenous ligands (Table 1). To verify the quality of the 3D models, the Ramachandran Plot Server was used. The obtained results showed that more than 90% of the residues in both structures were in the favored regions.

#### 3.5.5. Analysis of Sialic Acid-S1 Protein Interaction by Molecular Docking

**Figure 6:**
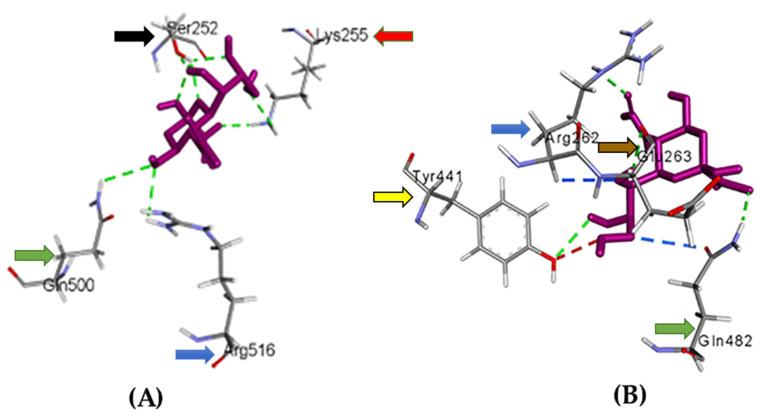
Sialic acid interacted with mutant (**A**) and wildtype (**B**) S1 proteins. Visualization of molecular docking outputs was performed by Discovery Studio. Ligand (sialic acid) was colored purple, while bond interactions were indicated with different colors. The green color refers to hydrogen bonds, blue color shows carbon-hydrogen bonds, and red color refers to acceptor hydrogen bonds. Residues of aa included in the interactions were indicated with an ID and three letters. Black arrow refers to serine, green arrow indicates glutamine, blue arrow shows arginine, red arrow refers to lysine, yellow arrow shows tyrosine, and brown arrow refers to glutamic Acid.

It has been shown that sialic acid residues on cell surfaces have a major role in initiating IBV infection, particularly in the early stages [17,18,19]. Therefore, molecular docking was employed to detect the binding affinity of S1 proteins to sialic acid residues. The binding affinity of sialic acid to the mutant S1 protein was −5.8 kcal/mol, while the wildtype S1 protein had −6 kcal/mol, so the mutant S1 protein binds to sialic acid with lower stability. Visualization of the docking outputs using Discovery Studio showed some differences in the mutant and wildtype S1 proteins (Figure 6). In terms of binding sialic acid with mutant S1 protein, there were seven hydrogen bonds, in which three bonds were located at residue 252, two bonds at residue 255, and finally two bonds at aa residues 500 and 516. While wildtype S1-sialic interactions revealed four hydrogen bonds at aa residues 262, 263, 441, and 482, two carbon-hydrogen bonds were detected at aa residues 262 and 482. Lastly, there was an acceptor hydrogen bond at aa 441.

## 4. Discussion

Over the last few years, DMV/1639 became the most prevalent strain of IBV in the Eastern part of Canada. The IBV S1 gene plays a major role in virus evolution and the emergence of variants. Our study was conducted to analyze the whole genome sequence of an IBV isolate (IBV/Ck/Can/2558004) and to identify the evolutionary trends within the S1 gene, with potential functional and structural impacts within the encoded protein. In the present study, the nt sequence of the S1 gene of IBV/Ck/Can/2558004 was almost identical (above 99%) to that of Canadian DMV/1639 IBV strains isolated in 2019 [24] and was less similar (96.63%) to the S1 gene of US DMV/1639 IBV isolated in 2011 [23]. Homology analysis of aa sequences revealed aa substitutions, predominantly in the HVRs of the IBV/Ck/Can/2558004 S1 protein compared to that of US DMV/1639. These aa changes in the S1 protein of IBV/Ck/Can/2558004 could result in potential functional and structural impacts.

Our results revealed that IBV/Ck/Can/2558004 had 27, 710 nt long, consisting of 13 ORFs organized as 5′UTR-1a-1b-S-3a-3b-3c(E)-M-4b-4c-5a-5b-N-6b-3′UTR, and this agreed with previously characterized IBV isolates in some parts of the world [74,75]. Additionally, our findings showed the presence of a non-coding region consisting of eight nt located between genes N and 6b, which has been previously recognized [76].

In the current work, the S1 cleavage site of IBV/Ck/Can/2558004 was RRSRR, and was thus in accordance with a similar study [77]. The homology analysis based on the S1 gene sequences revealed that IBV/Ck/Can/2558004 shared the highest nt identity percentage (99.44–99.63%) in comparison with five Canadian DMV/1639 IBV isolates recently isolated from Eastern Canada [24]. In addition, based on S1 sequences, nt similarity between IBV/Ck/Can/2558004 and two DMV/1639 IBV isolates, MDL_DMV1639_15-5582 and GA9977/2019, were 96.63% and 97.69%, respectively; these two IBV strains were previously isolated in the USA in 2011 and 2019, respectively [23,73]. Based on S1 nt sequences, our findings showed that IBV/Ck/Can/2558004 belonged to the GI-17 genotype that contains IBV strains isolated in the USA with different clinical diseases [22]. Transmission of IBV variants to Canada, which were previously characterized in the USA, could be attributed to close geographic distance as well as the common trade between the two countries [78].

With regards to the alignment of aa sequences in the S1 protein, IBV/Ck/Can/2558004 had 29 aa substitutions that represented 5.22% aa dissimilarity when aligned with that of the US DMV/1639 (MDL_DMV1639_15-5582), while it shared only 2.8% and 0.75 to 1.12% aa difference compared to the other US DMV/1639 (GA9977/2019) and the five Canadian DMV/1639 IBVs, respectively. Interestingly, it has been observed that a reasonable numbers of these aa substitutions were located within and/or near the three HVRs of the S1 subunit, thus reflecting a continuous change of neutralizing sites and serotype-specific epitopes with subsequent lower protective efficacy of homologous IB vaccines. These observations agreed with other previous studies, which indicated that the different serotypes within the genetically divergent IBVs are generally associated with changes in the HVRs of the S1 subunit [79,80]. Another study showed that the emergence of a new IBV serotype could have resulted from 10 to 15 aa substitutions (represent 2 to 3%) within the S1 glycoprotein [81]. Paradoxically, diversity within the IBV S1 gene are not often associated with new antigenic variants [82,83].

For N-glycosylation sites, our findings showed a lack of 3 N-glycosylation sites within the S1 protein of IBV/Ck/Can/2558004 compared to that of US DMV/1639 (MDL_DMV1639_15-5582). One of the three lost N-glycosylation sites was detected at position 267 because the consensus N-glycosylated sequence (Asn-X-Ser/Thr) was lost because the aa residue I substituted T at position 269. These changes in the N-glycosylation sites may have some functional consequences in the S1 protein. Similar analyses showed that the differences in N-glycosylation motifs of viral proteins could influence the receptor binding capacity and therefore minimize the recognition capacity by host innate immune responses and antibodies, ultimately affecting viral infectivity and replication [31,32,33]. Previous studies were conducted to analyze the N-glycosylation sites within the S1 glycoprotein of different IBV strains; their observations also revealed loss or acquisition of N-glycosylation sites in the S1 subunit of the compared IBV strains [84,85]. In terms of potential phosphorylation sites, the S1 protein of IBV/Ck/Can/2558004 revealed loss and acquisition in comparison with that of US DMV/1639 (MDL_DMV1639_15-5582) at certain positions; these observations were in accordance with previous studies [84,85]. Wilbur et al. [86] pointed out that the property of phosphorylation could be observed only in the N protein of coronaviruses. However, we observed potential phosphorylation motifs within the S1 glycoprotein based on the predictive tool, but this has no consequence for phosphorylation because S is an established glycoprotein that does not possess this property. Therefore, further investigations are warranted to confirm whether the S glycoprotein has phosphorylation peptides similar to those of the N protein. Our observations on palmitoylation sites within the S1 subunit demonstrated no substantial distinctions between the two tested IBV isolates; these findings contradicted other experimental studies that showed mutations in the palmitoylation activity within different viral proteins [84,87,88,89].

It has been shown that virus evolution is associated with two essential steps: the appearance of genetic variations and selection [90]. Here, we observed that 35 aa (6.5%) within the S1 subunit were subjected to positive selection pressure, and some of them were present in or near the three HVRS, which is in accordance with an experimental study on Italy 02 IBV isolates, which revealed evidence of aa residues with positive selection pressure among the HVRs [91]. The occurrence of positive selection pressure in the S1 protein of Canadian DMV/1639 IBV is not suggested to be driven by vaccine-induced immune pressure; there are two reasons for this: (1) no available homologous commercial vaccines against DMV/1639 in Canada, (2) the birds are often exposed to a heterologous immune response against that isolate. A previous study supported our suggestion, which revealed that vaccine-driven immune pressure could enhance the selective forces within the S1 protein of QX strains, particularly after administration of a homologous vaccine [92]. However, the positive selection pressure could be attributed to other factors beyond the resistance of the host, such as the biosecurity measures [90,93]. On the other hand, the partially immunized birds, because of unsuitable vaccination and immunosuppressive agents, could enhance the virus circulation, resulting in the selection and emergence of newly immune-evaded variants [94,95].

We employed further bioinformatic tools to predict if aa changes had an influence on the function, structure, and protein–ligand interaction in the S1 glycoprotein of IBV/Ck/Can/2558004 compared to that of US DMV/1639. Occasionally, the bioinformatic analysis could output negative results; however, we overcame this bias by using several tools. Initially, seven tools were used to determine the functional impacts. Eight variant aa residues (L2S, Q31R, G116H, T269I, G520D, Q522H, F523H, N526G) out of the substituted ones (29 aa) were unanimously detected by four tools to functionally affect the S1 subunit of IBV/Ck/Can/2558004. The eight filtered variants were then involved in the following analyses. It was shown that two mutants (G116H and T269I) were collectively detected by all tools to structurally impact the S1 subunit; however, the other mutants were predicted to influence the protein structure by some tools. The functional and structural consequences within the S1 subunit of IBV/Ck/Can/2558004 may alter the binding affinity to its natural receptors (sialic acid). Therefore, we performed molecular docking to identify the interactions between S1 glycoprotein and its relevant ligand. It was notable that the binding energy of the S1 glycoprotein of IBV/Ck/Can/2558004 to the sialic acid was lower compared to that of US DMV/1639. The possible explanation for this scenario would be that the S1 subunit contains two separate folded domains: the N-terminal domain (NTD) (aa 21 to 237) and the C-terminal domain (CTD) (aa 269 to 414), which act as the receptor binding domain [96]. Depending on the later observation, our findings showed that the most impactful aa residues on the S1 subunit function and structure were clustered in the same sequences of both domains (NTD and CTD). Ultimately, the occurrence of mutations within these domains could result in decreased binding potency to the ligands. Another experimental analysis was conducted on the M41 spike protein; it was shown that the aa sequence from 19 to 272 is necessary for S protein binding to the trachea as well as attachment to 2, 3-linked sialic acid [97].

The forementioned in silico findings reflect continuous changes in the S1 gene and its encoded protein of IBV/Ck/Can/2558004; subsequently, this may affect the virus pathogenesis and also lead to the existence of dozens of serotypes, which are problematic with regard to vaccination efficiency and could result in a high prevalence of Canadian DMV/1639 among Canadian poultry operations. The data obtained by the Animal Health Lab (University of Guelph, ON, Canada) from 2015 to 2016 supported our findings, in which the prevalence of DMV/1639 had reached to just below 25% among the detected IBV strains in the country in 2016. In 2017 and 2018, there was a substantial increase in the prevalence of DMV/1639, representing around 45% of all IBV strains in Canada, which dominated all other strains [98]. Furthermore, a recent vaccine efficacy study revealed that the Mass and Connecticut (Conn) vaccines could not provide complete protection against DMV/1639 in specific pathogen-free layer chickens [99]. Therefore, there is a strong probability that the higher rates of genetic diversity within the DMV/1639 S1 gene, together with lower vaccine efficacy, may lead to a further increased prevalence of Canadian DMV/1639 in chicken flocks in Canada.

In conclusion, our results elucidated that the S1 glycoprotein of Canadian DMV/1639 has experienced remarkable evolutionary changes in the form of mutations and selection pressure, which lead to functional and structural impacts within this protein. It is important to note that the S1 glycoprotein possesses a significant role in cell binding and receptor identification, and genetic diversity is frequently observed in this protein of IBV. Therefore, by identifying these properties, we will be able to better understand the epidemiology, the molecular mechanisms of infection, and virus evolution. Additionally, the continuous evolution of the S1 glycoprotein of Canadian DMV/1639 indicates the significance of constant monitoring of circulating IBV strains and the necessity of developing effective vaccines that counteract the emergence of new variants. The approach of computational analysis is user-friendly, reliable, accessible, and essential to predict multiple events within a gene. However, validation of these predictions requires proper laboratory and in vivo experiments.

## Figures and Tables

**Table 1:** TM- and C-scores, and predicted aa residues bound to exogenous ligand (magnesium).

Sequences	C-Score	TM-Score	Ligand Binding Site Residues	Ligand
Mutant S1 protein	0.19	0.74 ± 0.11	232,235,236	Magnesium
Wildtype S1 protein	−0.17	0.69 ± 0.12	232,235,236	Magnesium

## Data Availability

The datasets used and/or analyzed within the frame of the study can be provided by the corresponding author upon reasonable request.

## Supplementary Materials

The following supporting information can be downloaded at: https://www.mdpi.com/article/10.3390/genes13091617/s1, Table S1: Eighty-five reference IBV sequences used in phylogenetic analysis based on S1 gene nucleotide sequence; Table S2: S1 glycoproteins of IBV/Ck/Can/2558004, Canadian, and US DMV/1639 IBVs utilized in pairwise alignment; Table S3: Percent S1 amino acid sequence similarity of IBV/Ck/Can/2558004, Canadian, and US DMV/1639 IBVs; Table S4: Prediction of conserved serine, threonine, and tyrosine phosphorylation sites in S1 glycoprotein of IBV/Ck/Can/2558004 and MDL_DMV1639 _15-5582 (KX529725); Table S5: Prediction of potential palmitoylation sites in S1 glycoprotein of IBV/Ck/Can/2558004 and MDL_DMV1639 _15-5582 (KX529725).
