# Supplementary material for: Genotyping and In Silico Analysis of Delmarva (DMV/1639) Infectious Bronchitis Virus (IBV) Spike 1 (S1) Glycoprotein
Source: Genes (Basel). 2022 Sep 9;13(9):1617. doi: 10.3390/genes13091617 (PMC9498812; doi:10.3390/genes13091617)
Supplement: Supplementary file 1 [file genes-13-01617-s001.zip › genes-1869495-supplementary.pdf]

Supplementary Table S1. 85 reference IBV sequences used in phylogenetic analysis based on S1 gene nucleotide sequence.

Reference sequence	Accession number	Country
N1/08	JN176213	Australia
V18/91	U29521	Australia
N4/02	DQ059618	Australia
N5/03	DQ059619	Australia
B1648	X87238	Belgium
IBV/Brasil/351/1984	GU393339	Brazil
UFMG/1141	JX182783	Brazil
Qu_mv	AF349621	Canada
Qu16	AF349620	Canada
IBV/Ck/Can/17-035614	MN512434	Canada
IBV/Ck/Can/17-036989	MN512435	Canada
IBV/Ck/Can/18-048192T	MN512436	Canada
IBV/Ck/Can/18-048430	MN512437	Canada
IBV/Ck/Can/18-049707	MN512438	Canada
48SD-96VI	KC577388	China
QXIBV	AF193423	China
CK/CH/LLN/111169	KF411040	China
Connecticut vaccine	KF696629	China
QS	JQ250818	China
SDW	DQ070840	China
SDIB781/2012	KF007209	China
TC07-2	GQ265948	China
ck/CH/LHLJ/110664	JQ739299	China
GX2-98	AY251816	China
ck/CH/LSD/110857	JQ739375	China
4/91 vaccine	KF377577	China
ck/CH/LSD/110712	JQ739363	China
CK/CH/LDL/97I	EF030995	China
Q1	AF286302	China
Eg/1265B/2012	KC533682	Egypt
FR-85131-85	AJ618985	France
It/497/02	DQ901377	Italy
IZO 28/86	KJ941019	Italy
IBV422	KF809791	India
V25	KF757451	India
Variant 1	AF093795	Israel
IS/1201	DQ400359	Israel
Variant 2	AF093796	Israel
JP8443	AY296745	Japan
K620/02	FJ807944	Korea
SNU8067	JQ977697	Korea
A	AF151953	New Zealand

D	AF151956	New Zealand
K43	AF151958	New Zealand
H120	FJ888351	The Netherlands
D274	X15832	The Netherlands
BL-56	AF352831	Mexico
Moroccan-G/83	EU914938	Morocco
NGA/N544/2006	FN182269	Nigeria
NGA/324/2006	FN182277	Nigeria
NGA/295/2006	FN182276	Nigeria
NER/28/2007	FN182272	Niger
RF/01/02	AJ441314	Russia
Spain/00/336	DQ386098	Spain
Spain/98/313	DQ064808	Spain
TP/64	AY606320	Taiwan
UK/L-633/04	DQ901376	United Kingdom
UK/7/91	Z83975	United Kingdom
6/82	X04723	United Kingdom
Beaudette	M95169	USA
M41	AY561711	USA
Conn46 1996	FJ904716	USA
Iowa97	GU393337	USA
Gray	L14069	USA
JMK	L14070	USA
PA/5344/98	AY789947	USA
Holte	L18988	USA
L905	JQ964070	USA
SE17	M99484	USA
ArkDPI	AF006624	USA
Arkansas Vaccine	GQ504721	USA
CAL99	DQ912831	USA
PA/Wolgemuth/98	AF305595	USA
AL/6609/98	AF510656	USA
MDL_DMV1639 15-6636	KX529720	USA
MDL DMV1639 15-5582	KX529725	USA
CA/1737/04	EU925393	USA
DMV/5642/06	EU694402	USA
GA/13485/2013	KP085597	USA
GA/13384/2013	KM660635	USA
GA08	GU301925	USA
DE/072/92	U77298	USA
GA/13055/00	AF338719	USA
CU82792	AF317214	USA
GA/19010/19	MK570452	USA

Supplementary Table S2. S1 glycoproteins of IBV/Ck/Can/2558004, Canadian, and US DMV/1639 IBVs utilized in pairwise alignment.

Reference sequence	Accession number	aa*	Country	Year of isolation
IBV/Ck/Can/17-035614	MN512434	536	Canada	2019
IBV/Ck/Can/17-036989	MN512435	536	Canada	2019
IBV/Ck/Can/18-048192T	MN512436	536	Canada	2019
IBV/Ck/Can/18-048430	MN512437	536	Canada	2019
IBV/Ck/Can/18-049707	MN512438	536	Canada	2019
MDL_DMV1639 15-6636	KX529720	537	USA	2011
MDL DMV1639 15-5582	KX529725	537	USA	2011
GA/19010/19	MK570452	537	USA	2019

aa*: The number of amino acids in the S1 protein sequence.

Supplementary Table S3. Percent S1 amino acid sequence similarity of IBV/Ck/Can/2558004 (bold), 5 Canadian DMV/1639 IBVs (shaded) and 3 US DMV/1639 IBV isolates (non-shaded).

		1	2	3	4	5	6	7	8	9
**1**	**IBV/Ck/Can/2558004**		99.25%	99..07%	99.07%	98.88%	99.07%	97.20%	94.78%	94.78%
**2**	IBV/Ck/Can/17-035614	99.25%		99.63%	99.44%	99.25%	99.44%	97.20%	95.52%	95.52%
**3**	IBV/Ck/Can/18-048430	99.07%	99.63%		99.63%	99.07%	99.25%	97.20%	95.15%	95.15%
**4**	IBV/Ck/Can/18-048192T	99.07%	99.44%	99.63%		99.07%	99.25%	97.20%	94.96%	94.96%
**5**	IBV/Ck/Can/17-036989	98.88%	99.25%	99.07%	99.07%		99.81%	96.83%	95.15%	95.15%
**6**	IBV/Ck/Can/18-049707	99.07%	99.44%	99.25%	99.25%	99.81%		97.02%	95.34%	95.34%
**7**	GA/19010/19	97.20%	99.20%	97.20%	97.20%	96.83%	97.02%		95.53%	95.53%
**8**	MDL_DMV1639_15-5582	94.78%	95.52%	95.52%	94.96%	95.15%	95.34%	95.53%		100%
**9**	MDL_DMV1639_15-6636	94.78%	95.52%	95.52%	94.96%	95.15%	95.34%	95.53%	100%	

Supplementary Table S4. Prediction of conserved serine (red, underlined), threonine (blue, underlined), and tyrosine (green, underlined) phosphorylation sites in S1 glycoprotein of IBV/Ck/Can/2558004 and MDL_DMV1639 _15-5582 (KX529725). The positions are denoted with superscripts.

Serine phosphorylation sites	Threonine phosphorylation sites	Tyrosine phosphorylation sites
YYYQS^{32}AFRP	LLLVT^{10}PLFA	SAVLY^{21}DNNS
FRPGS^{38}GWHL	VSEQTSPGT^{56}	HGGAY^{47}AVVN
SEQTS^{57}PGTC	SVAMT^{80}APLN	IEQNY^{128}IRIA
AIGYS^{69}MNFS	RSNGT^{139}GPRD	NETLY^{185}VSGA
AWSGS^{91}QFCT	FYNST^{149}VSVS	AQSGY^{306}YNFN
LFYNS^{148}TVSV	DLVFTSNET^{179}	SDFMY^{326}GSYH
NSTVS^{151}VSKY	T^{183}SNETLYVS	ISLAY^{355}GPLQ
TVSVS^{153}KYPK	GVDFT^{194}AGGP	CCYAY^{377}SYNG
PFTNS^{251}SLVK	GGPIT^{200}YKVM	KCVDY^{438}NIYG
YRENS^{265}VNTT	LCDGT^{227}PRGL	DYNIY^{441}GRVG
VTAQS^{304}GYYN	QTYQT^{299}VTAQ	ENQFY^{529}IKLT
VYKAS^{322}DFMY	SRIQT^{415}TTKP	
FMYGS^{328}YHPK	RIQTT^{416}TKPH	
LWFNS^{348}LSIS	IQTTT^{417}KPHV	
FNSLS^{350}ISLA	PHVLT^{423}THFY	
GCKQS^{365}VFNG	HVLTT^{424}HFYN	
CYAYS^{378}YNGP	QGFIT^{450}NVTD	
KSDGS^{411}RIQT	VGILT^{514}SRNV	
NVTDS^{455}TADY		
GILTS^{515}RNVT		
NVTGS^{521}QFLE		

Supplementary Table S5. Prediction of potential palmitoylation sites (red color) in S1 glycoprotein of IBV/Ck/Can/2558004 and MDL_DMV1639 _15-5582 (KX529725).

Peptide	Position	Score	Cut-off	Cluster
VTLLFALCSAVLYDN	16	29	0	Cluster C
AQTSPGSCTAGAIGY	61	7.572	0	Cluster A
SWSDSQFCTAHCNFT	94	1.754	0	Cluster B
SQFCTAHCNFTNIVV	98	6.645	0	Cluster A
IVVFVTHCFKRGQHF	110	2.012	0	Cluster C
FKRGQHFCPLTGFIE	118	4.514	0	Cluster A
PKFRSLQCVNNQTSV	163	3.797	0	Cluster A
TAHDVILCDGTPRGL	224	12.797	0	Cluster A
TPRGLLACQYNTGNF	234	1.996	0	Cluster C
YGSYHPKCNFRPETL	333	2.408	0	Cluster C
YGPLQGGCKQSVFNN	362	2.126	0	Cluster C
VFNNRATCCYAYSYN	373	7.359	0	Cluster B
FNNRATCCYAYSYNG	374	2.757	0	Cluster C
SYNGPRACKGVYRGQ	385	1.429	0	Cluster C
QLQQLFECGLLVYIT	399	4.314	0	Cluster A
NNITLDKCVDYNIYG	435	4.584	0	Cluster A
NFYKVNPCEDVNQQF	495	1.243	0	Cluster C
